# Immunogenicity of an *Escherichia coli*-Produced Recombinant 9-Valent Human Papillomavirus Vaccine in Mice and Rats

**DOI:** 10.3390/vaccines14050407

**Published:** 2026-05-01

**Authors:** Yu-Ying Liu, Fei Yin, Wen-Juan Li, Dan Chen, Shu-Ming Wu, Xiao Chen, Yan Wang, Zeng-Min Yang, Hai-Jiang Zhang, Yong-Jiang Liu

**Affiliations:** Beijing Health Guard Biotechnology Inc., Beijing 100176, China; yuy.liu@hotmail.com (Y.-Y.L.); f.yin@klws.com (F.Y.); wj.li@klws.com (W.-J.L.); d.chen@klws.com (D.C.); sm.wu@klws.com (S.-M.W.); x.chen@klws.com (X.C.); y.wang@klws.com (Y.W.); jl.xue@klws.com (Z.-M.Y.)

**Keywords:** human papillomavirus, vaccine, immunogenicity, immune persistence, *Escherichia coli*

## Abstract

**Background:** Prophylactic human papillomavirus (HPV) vaccines are crucial for preventing HPV-related cancers. This study aimed to preclinically evaluate a novel recombinant 9-valent HPV vaccine produced in *Escherichia coli* (*E. coli*), which targets HPV types 6, 11, 16, 18, 31, 33, 45, 52, and 58, and is based on virus-like particles (VLPs) of the HPV major capsid protein L1. **Methods:** The molecular weight and purity of HPV L1 protein bands were assessed by sodium dodecyl sulfate-polyacrylamide gel electrophoresis (SDS-PAGE) with Coomassie Brilliant Blue staining. The morphology and size distribution of VLPs were characterized using cryo-electron microscopy and DLS. The immunogenicity and durability of the recombinant 9-valent HPV vaccine were evaluated in BALB/c mice and Wistar rats. Mice received single or triple immunizations (2-week intervals) of two vaccine batches or Gardasil^®^9 (MSD, USA) control at 1/20 human dose. Antibody responses were monitored via ELISA and pseudovirus neutralization assays over 24 weeks. Rats were administered single or triple immunizations (2-week intervals) of high- (1/10), medium- (1/20), or low-dose (1/40) vaccine or Gardasil^®^9 control (1/20), with neutralizing antibodies tracked for 16 weeks. **Results:** Cryo-electron microscopy and DLS revealed that VLPs of each type appeared as uniformly distributed, spherical or ellipsoidal hollow intact particles with a diameter of approximately 45–65 nm. This vaccine demonstrated robust immunogenicity and long-lasting efficacy in BALB/c mice and Wistar rats, with effects comparable to those of the commercially available vaccine Gardasil^®^9. **Conclusions:** The 9-valent HPV vaccine induces robust and persistent immune responses in mice and rats, strongly supporting further clinical trials. It is expected to be an alternative to marketed vaccines and ease the global supply shortage of 9-valent HPV vaccines.

## 1. Introduction

HPV constitutes a group of DNA viruses with a tropism for infection of the epithelial cells of human skin and mucous membranes. It is transmitted predominantly through sexual contact, but also via close skin-to-skin contact or through vertical transmission from mother to child [1,2]. To date, over 200 distinct HPV types have been identified [3]. Clinically, these are categorized into two groups based on their pathogenic potential. Low-risk HPV types, such as HPV 6 and 11, are primarily associated with benign proliferations like cutaneous warts and anogenital warts. High-risk types, most notably HPV 16 and 18, are the primary causative agents for several cancers. Persistent infection with these high-risk types is etiologically linked to cervical cancer, as well as other anogenital cancers (e.g., anal, penile, vulvar, vaginal) and a subset of oropharyngeal cancers [2,3,4].

The global cancer burden attributable to HPV is substantial and underscores its status as a critical public health issue. Annually, an estimated 625,600 women and 69,400 men are diagnosed with HPV-related cancers globally. Cervical cancer represents the most significant manifestation of this burden, accounting for 93% of HPV-related cancers in women, and ranking as the fourth leading cause of female cancer incidence and mortality in 2020 [5,6].

Vaccination is the most cost-effective strategy to prevent HPV transmission. Prophylactic HPV vaccines primarily induce the host’s humoral immune response, producing neutralizing antibodies that bond with the virus antigen upon HPV entry into the cell, thereby preventing HPV infection. By preventing initial HPV infection and reducing persistent HPV infection, the development and progression of precancerous cervical lesions can be blocked. Despite the existence of effective preventive tools, high incidence of and mortality from HPV-induced cervical cancer persist, largely driven by low global coverage of both HPV vaccination and cervical screening. Particularly in developing countries, vaccine cohorts are primarily women aged 18–45, while the majority of girls aged 9–14 remain unvaccinated due to high vaccine prices, limited supplies, and insufficient awareness of the vaccine’s preventive benefits [7,8,9]. In response to this ongoing crisis, the World Health Organization (WHO) formally launched the landmark “Global Strategy to Accelerate the Elimination of Cervical Cancer” in November 2020. This strategy defines ambitious “90-70-90” goals for 2030. That is, 90% of girls receive the HPV vaccine before the age of 15, 70% of women undergo efficient cervical cancer screening before the ages of 35 and 45, and 90% of women with precancerous cervical conditions and cervical cancer receive timely treatment [10,11].

The global demand for HPV vaccines has escalated dramatically, fueled by the WHO elimination strategy and expanding national introductions. As of November 2024, 147 WHO member countries had introduced HPV vaccination in their routine immunization programs. Global coverage of the first HPV vaccine dose among girls was estimated at 31%, representing a substantial increase from 17% in 2019 and 27% in 2023 [12]. China officially included the HPV vaccine in its national immunization program in November 2025, providing free vaccination for 13-year-old girls [13]. The global human papillomavirus vaccines market was estimated at USD 5.4 billion in 2024. The market is expected to grow to USD 14.1 billion in 2034 [14]. According to MI4A, the estimated global demand for HPV vaccines is expected to reach about 140 million doses by 2026 and stabilize at about 125 million doses by 2031 after completing Multi-Age Cohorts (MACs) immunization activities [15]. Such large-scale initiatives underscore the urgent need to expand affordable vaccine supply.

Globally, six prophylactic HPV vaccines have been approved: the quadrivalent Gardasil^®^ [16], the bivalent Cervarix^®^ [17], the 9-valent Gardasil^®^9 [18,19], the bivalent Cecolin^®^ [20], the bivalent Walrininvax^®^ [21] and the 9-valent Cecolin^®^9 [22] (first licensed in 2006, 2007, 2014, 2019, 2022, and 2025, respectively). In 2023, the Serum Institute of India’s quadrivalent vaccine, Cervavac [23], entered the WHO prequalification process. All these vaccines target HPV types 16 and 18, which are responsible for approximately 71% of cervical cancer cases worldwide. The quadrivalent and 9-valent vaccines also cover HPV types 6 and 11, which cause over 90% of genital warts. Additionally, the 9-valent vaccine includes five high-risk types—HPV 31, 33, 45, 52, and 58—which, together with types 16 and 18, can prevent around 90% of cervical cancer cases globally [24,25,26].

Nevertheless, current vaccine supply falls short of global demand. All licensed HPV vaccines are based on VLPs assembled from the L1 capsid protein. The Gardasil series and Walrinvax^®^ use yeast expression systems, while Cervarix^®^ uses a baculovirus–insect cell system. These platforms are effective but expensive and difficult to scale, perpetuating supply shortages [27]. Gardasil^®^9, with the broadest coverage, faces especially high demand and limited availability in low-income, high-prevalence regions. The Cecolin series, produced in *Escherichia coli* [20,22], offers a more cost-effective alternative but still cannot meet the overwhelming global need. The supply gap for affordable, broadly protective vaccines remains vast.

To address the dual challenges of broad-spectrum protection and scalable, affordable production, we have developed a novel 9-valent HPV VLP vaccine candidate targeting types 6, 11, 16, 18, 31, 33, 45, 52, and 58 [28]. It utilizes an *E. coli* expression system, chosen for its potential for high yield and cost-efficiency. While other 9-valent HPV vaccine candidates in preclinical or clinical development are predominantly manufactured using eukaryotic expression systems (e.g., yeast or insect cells), only a small number are produced in *Escherichia coli*. Our candidate employs truncated L1 proteins that form pentamers in vivo, which subsequently self-assemble into VLPs after purification, obviating the need for in vitro pentamer folding steps required in the manufacturing process of other *E. coli*-produced vaccines.

This study is designed to conduct a preclinical evaluation of this candidate vaccine, including the analysis of its particle characterization, and assessment of immunogenicity and the durability of the immune response in mice and rats. This foundational work is critical for establishing proof-of-concept and guiding future clinical development. This candidate 9-valent HPV vaccine is expected to elicit robust and long-lasting immune responses in these two preclinical animal models, with levels superior or at least non-inferior to those of the commercially available Gardasil^®^9, supporting its potential as a viable alternative to currently marketed HPV vaccines.

## 2. Materials and Methods

### 2.1. Vaccines

Our 9-valent HPV vaccine was prepared as described previously [28]. Particularly, this vaccine is constituted from the pentamers of the main structural protein L1 of HPV types 6, 11, 16, 18, 31, 33, 45, 52, and 58, purified and assembled in vitro into VLPs, and combined with an aluminum hydroxide adjuvant. This vaccine presents as a milky white suspended solution. Each 0.5 mL contains L1 VLPs of different HPV types: 30 mg (type 6), 40 mg (type 11), 60 mg (type 16), 40 mg (type 18), 20 mg (types 31, 33, 45, 52, and 58). The total quantity of antigens is 270 mg, and it contains an aluminum hydroxide adjuvant of 0.75 mg (as aluminum).

The positive control was Gardasil^®^9, a commercially available HPV 9-valent vaccine against HPV types 6, 11, 16, 18, 31, 33, 45, 52, and 58 (MSD, Rahway, NJ, USA).

### 2.2. SDS-PAGE

A 10% separation gel was selected and placed in the electrophoresis chamber, followed by the addition of 1× diluted electrophoresis buffer. Samples were loaded into the wells in sequence: 16 μL for experimental samples and 5 μL for molecular weight markers. Electrophoresis was initiated at a constant current of 10 mA per gel. The current was increased after the proteins entered the separation gel and continued until the bromophenol blue tracking dye migrated to the edge of the gel. Following electrophoresis, the gel was stained with Coomassie Brilliant Blue and destained with copious amounts of purified water or destaining solution until the background became nearly transparent. The gel was then scanned using a UVP GDS 8000 gel imaging analysis system (Bio-Rad, Hercules, CA, USA). Molecular weights were calculated based on band migration distances, and the purity of the sample was assessed using the area normalization method.

### 2.3. Dynamic Light Scattering (DLS)

DLS measurements were performed using a Malvern Nano ZS analyzer (Malvern Panalytical, Malvern, UK) to analyze the particle size by assessing Brownian motion of particles in sample solutions. A minimum volume of 800 μL of each sample solution was carefully pipetted into a dedicated cuvette. The particle size was determined by calculating the average value from three replicate measurements.

### 2.4. Cryo-Electron Microscopy

Cryo-electron microscopy images were captured using an FEI Tecnai Spirit (120 kV) (FEI, Hillsboro, OR, USA). Samples were negatively stained and then observed. The method of negative staining is as follows: approximately 3 mL of the sample was deposited to lie on a copper grid coating (Cu-200CN) for about 1 min. One drop of pure water was placed on the copper grid, and excess fluid was absorbed using filter paper. This procedure was repeated three times. Negative staining was carried out using 1% uranyl formate and the surplus liquid was again absorbed using filter paper. After repeating the washing procedures, the sample was dried for examination through an electron microscope.

### 2.5. Ethics and Animal Care

BALB/c mice and Wistar rats were selected to evaluate vaccine immunogenicity. BALB/c mice serve as a standard immunization model, while Wistar rats enhance result translatability due to their physiological similarity to humans.

All animal experiments comply with ARRIVE (Animal Research: Reporting of In Vivo Experiments) guidelines [29] and guidance on the operation of the Animals (Scientific Procedures) Act 1986 [30]. The anesthesia and euthanasia of animals comply with the American Veterinary Medical Association (AVMA) Guidelines for the Euthanasia of Animals (2020) [31].

BALB/c mice and Wistar rats were obtained from Beijing Vital River Laboratory Animal Technology Co., Ltd. (Beijing, China), housed in SPF conditions with a 12 h light/dark cycle, and acclimatized for at least 3 days before experimentation. All animals were immunocompetent, non-genetically modified, and had no prior experimental history.

Experimental procedures were performed gently to minimize stress; CO_2_ euthanasia was used humanely. Daily monitoring of appearance, activity and pain signs, and weekly monitoring of body weight, were performed; humane euthanasia would be performed for severe weight loss, lethargy or infection. All animals remained in good general health throughout the study period.

Sample size was determined based on previous studies and 3R principles, with 10 animals per group. Animals were randomly assigned to groups via computer-generated numbers; no blinding was performed. Animals displaying severe weight loss, lethargy, or infection were excluded. No pre-specified exclusion criteria were applied to individual data points. All 10 animals per group were included in the final statistical analysis.

### 2.6. Immunogenicity Design in BALB/c Mice

To measure the immunogenicity and immune persistence in mice, our recombinant 9-valent HPV vaccine, positive control (Gardasil@9) and negative control (50 μg aluminum hydroxide adjuvant) were administered to female BALB/c mice (6 to 8 weeks old, n = 10 per group, total N = 60 in this experiment) via intramuscular injection at 1/20 of the human dose, either as a single immunization or triple immunization at 2-week intervals. Venous blood samples were collected from all mice for neutralization assays and ELISA at weeks 2, 4, 6, 8, 12, 16, 20, 24. The experimental unit was defined as a single animal.

### 2.7. Immunogenicity Design in Wistar Rats

To measure the immunogenicity and immune persistence of the test vaccine in rats, Wistar rats (8–9 weeks old, 180–220 g in weight) received 1/10 dose of the 9-valent HPV vaccine for the high-dose group, 1/20 dose for the medium-dose group, and 1/40 dose for the low-dose group. The dose of positive control group was 1/20 dose of Gardasil@9. A total of 50 μg aluminum hydroxide adjuvant was set as negative control. The immunization strategy employed either a single dose or three doses at 2-week intervals, via intramuscular injection. Each group consisted of 10 rats, and the total number of rats in this experiment was 100. Venous blood samples were collected from all rats for neutralization assays at weeks 2, 4, 6, 8, 12, 16. The experimental unit was defined as a single animal.

### 2.8. ELISA

The antigen of a specific HPV type was diluted to 5 mg/mL in phosphate buffer and added to 96-well plates at 100 μL/well, followed by overnight coating at 4 °C. The plates were inverted to discard the liquid and patted dry. Each well was then blocked with 200 μL of blocking buffer (containing 2% bovine serum albumin) at 25 °C for 1.5 h. After blocking, diluted animal serum and negative control serum were added, followed by horseradish peroxidase (HRP)-labeled secondary antibodies. Repeated washing steps were performed, and TMB chromogenic substrate was added to each well. The reaction was incubated in the dark until color development was complete, terminated by adding 50 μL of 1 M sulfuric acid per well. Absorbance at 450 nm (OD values) was measured using a microplate reader. Serum binding antibody titers were determined by comparing the mean OD values of the sample wells with negative control wells using the Reed–Muench method.

### 2.9. Pseudovirus Neutralization Assay

In a 96-well cell culture plate, 1.5 × 10^4^ 293 FT cells were added to each well and then incubated in a 37 °C, 5% CO_2_ incubator for 4–6 h. An equal volume of pseudovirus was added into each serially diluted serum sample well and control well on the sample plate. The plate was gently tapped to ensure thorough mixing and then incubated at 4 °C for 1 h. The serum–pseudovirus mixture was transferred to the corresponding cell plate where the 293 FT cells were seeded. Finally, the cell culture plate was placed in a 37 °C, 5% CO_2_ incubator for three additional days. Post-incubation, the number of fluorescent cells in each well was read by a CTL S6 Universal Analyzer (CTL, Shaker Heights, OH, USA). The required serum dilution for 50% reduction in fluorescence cell number in sample wells compared to control wells was calculated using the Reed–Muench method. This value was assigned as the neutralization titer of the serum.

### 2.10. Statistics

All statistical analyses were conducted using GraphPad Prism software (Version 9; GraphPad Software, La Jolla, CA, USA). Antibody titers were presented as the geometric mean with a 95% confidence interval (CI).

The Shapiro–Wilk test was used to evaluate normality. Normally distributed data were analyzed with parametric tests. Non-normally distributed data were analyzed using non-parametric methods.

For comparisons between two independent groups, statistical differences were assessed using multiple unpaired *t*-tests, and the Holm–Sidak correction method was applied for post hoc adjustment.

For comparisons among three or more independent groups, statistical differences were evaluated using one-way analysis of variance (ANOVA). Subsequent post hoc multiple comparisons were performed via Tukey’s honestly significant difference (HSD) test to identify specific pairwise differences between groups.

A *p*-value < 0.05 was defined as statistically significant. The significance levels were annotated as follows: not significant (ns, *p* > 0.05); *, *p* ≤ 0.05; **, *p* ≤ 0.01; ***, *p* ≤ 0.001; ****, *p* < 0.0001.

## 3. Results

### 3.1. Characterization of HPV VLPs

**Figure 1:**
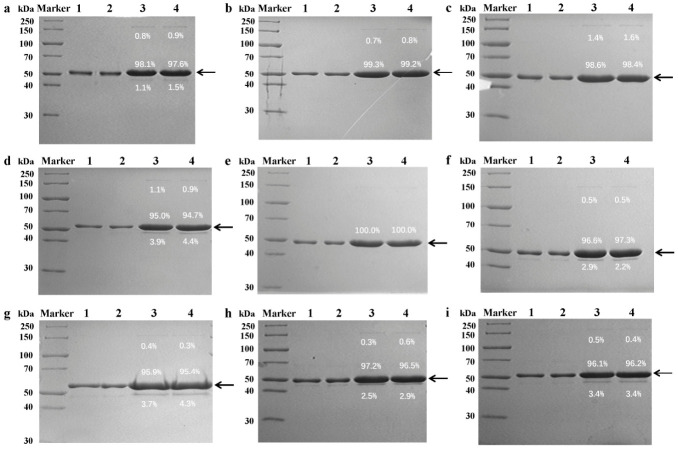
SDS-PAGE stained with Coomassie Brilliant Blue of HPV L1 proteins. Lanes 1 and 2 were loaded with 3 μg proteins to analyze molecular weights. Lanes 3 and 4 were loaded with 10 μg proteins to analyze purity. (**a**) HPV6. (**b**) HPV11. (**c**) HPV16. (**d**) HPV18. (**e**) HPV31. (**f**) HPV33. (**g**) lHPV45. (**h**) HPV52. (**i**) HPV58. Arrowheads indicate the bands of HPV L1 proteins. The percentage labeled beside each band represents its relative band intensity ratio. The original SDS-PAGE figures can be found in Supplementary Figure S1.

Truncated L1 proteins of HPV types 6, 11, 16, 18, 31, 33, 45, 52, and 58 were expressed in *E. coli*. Their quality was assessed via SDS-PAGE stained with Coomassie Brilliant Blue (Figure 1). The molecular weights of the major bands for each type were approximately: HPV6 at 51 kDa, HPV11 at 50 kDa, HPV16 at 49 kDa, HPV18 at 53 kDa, HPV31 at 48 kDa, HPV33 at 48 kDa, HPV45 at 56 kDa, HPV52 at 49 kDa, and HPV58 at 52 kDa, with purities all exceeding 90%.

**Figure 2:**
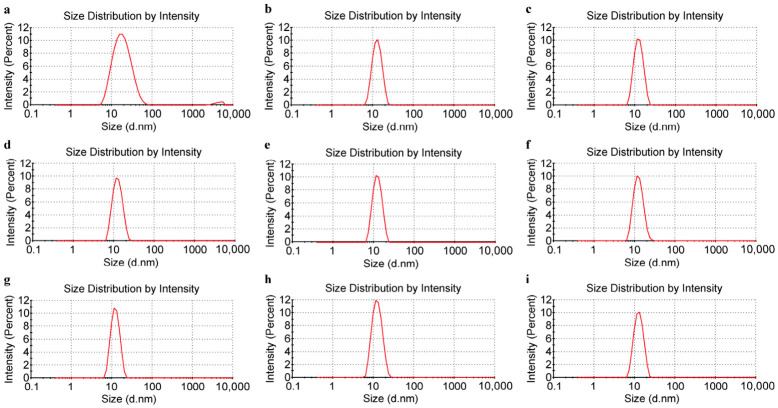
Size distribution of HPV L1 pentamers detected by DLS. (**a**) HPV6. (**b**) HPV11. (**c**) HPV16. (**d**) HPV18. (**e**) HPV31. (**f**) HPV33. (**g**) HPV45. (**h**) HPV52. (**i**) HPV58.

These truncated L1 proteins formed pentamers in *E. coli*. After purification of the lysate, the particle size and distribution of purified pentamers were tested by dynamic DLS (Figure 2), and the average particle sizes and polydispersity index (PdI) values are listed in Table 1. For HPV types 11, 16, 18, 31, 33, 45, 52, and 58, the particle sizes ranged from 12 to 13 nm, with narrow peak profiles and no impurity peaks. HPV6 exhibited a particle size of 16.7 nm, with a broader peak profile and significant impurity peaks, likely due to the slow self-assembly of its pentamers into larger particles.

**Figure 3:**
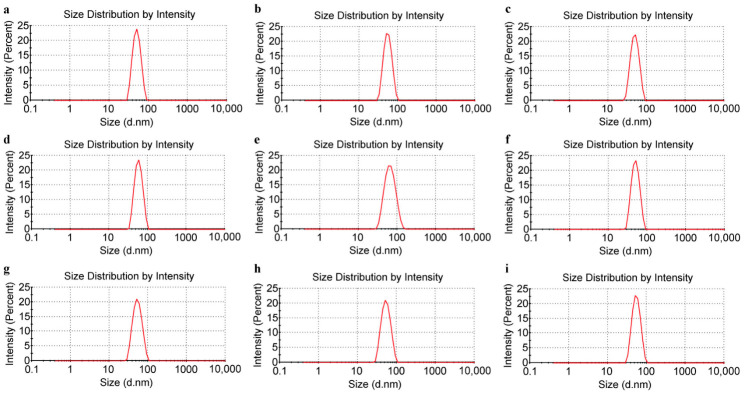
Size distribution of HPV L1 VLPs detected by DLS. (**a**) HPV6. (**b**) HPV11. (**c**) HPV16. (**d**) HPV18. (**e**) HPV31. (**f**) HPV33. (**g**) HPV45. (**h**) HPV52. (**i**) HPV58.

The particle size distribution of the L1 VLPs was analyzed using DLS (Figure 3), and the value results are shown in Table 1. The VLP sizes ranged from 45 to 65 nm, with PdI values all below 0.1. The low PdI value, narrow peak profiles and absence of impurity peaks indicated uniform VLPs.

**Figure 4:**
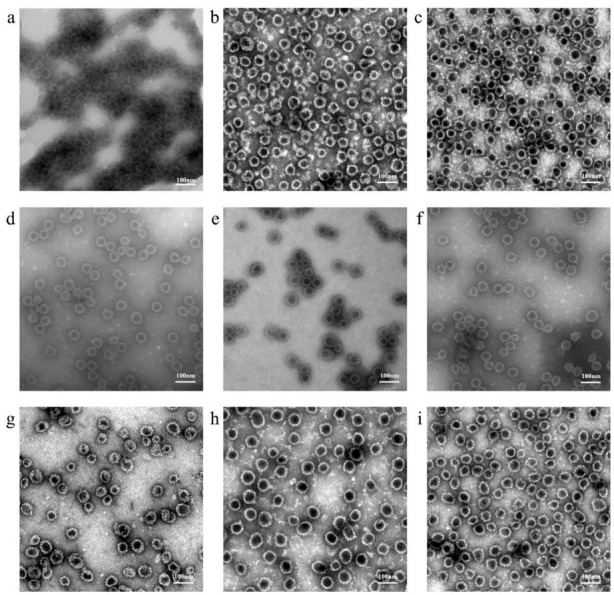
Cryo-electron micrographs of HPV VLPs. Scale bar, 100 nm. (**a**) HPV6 VLPs. (**b**) HPV11 VLPs. (**c**) HPV16 VLPs. (**d**) HPV18 VLPs. (**e**) HPV31 VLPs. (**f**) HPV33 VLPs. (**g**) HPV45 VLPs. (**h**) HPV52 VLPs. (**i**) HPV58 VLPs.

Morphological analysis of the purified L1 VLPs for each type was performed via cryo-electron microscopy (Figure 4), revealing uniformly distributed hollow intact particles with spherical or ellipsoidal shapes, measuring approximately 50–60 nm in diameter. These findings were consistent with the results from the DLS experiments.

### 3.2. The 9-Valent HPV Vaccine Elicited a Robust and Persistent Immune Response in Mice

To evaluate the immunogenicity of the test 9-valent HPV vaccine, female BALB/c mice (n = 10 per group) were immunized intramuscularly with three doses at 2-week intervals. The test vaccine was administered at 1/20 dose, with the licensed Gardasil^®^9 vaccine (1/20 dose) serving as a positive control. Serum samples were collected 2 weeks after the third immunization. Binding antibody levels were assessed by measuring serum HPV type-specific IgG titers via ELISA, and serum neutralizing antibody titers were quantified using a pseudovirus-based neutralization assay.

**Figure 5:**
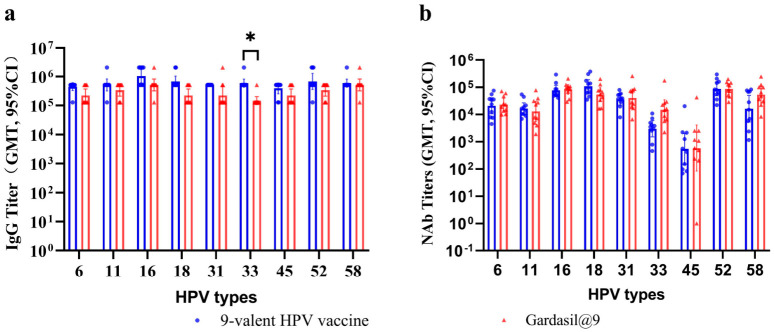
The IgG titers (**a**) and neutralizing antibody titers (**b**) in serum 2 weeks after the third vaccination in mice. BALB/c mice (10/group) were inoculated three times with 1/20 dose of the test 9-valent HPV vaccine or Gardasil@9. Error bars represent a 95% confidence interval. NAb, neutralizing antibody; *, *p* < 0.01.

For IgG antibody responses (Figure 5a), the test 9-valent HPV vaccine induced significantly higher IgG titers against HPV33 compared with Gardasil^®^9 (*p* = 0.048). No statistically significant differences in IgG titers were observed between the test vaccine and Gardasil^®^9 for the remaining eight HPV types (for HPV6, 11, 16, 18, 31, 45, 52, and 58; *p* = 0.133, 0.486, 0.242, 0.143, 0.800, 0.265, 0.242, and 0.866, respectively). Regarding neutralizing antibody responses (Figure 5b), the test vaccine elicited neutralizing antibody titers equivalent to those of Gardasil^®^9 across all nine HPV genotypes (for HPV6, 11, 16, 18, 31, 33, 45, 52, and 58; *p* = 0.997, 0.587, 0.589, 0.998, 0.997, 0.909, 0.596, 0.991, and 0.991, respectively).

**Figure 6:**
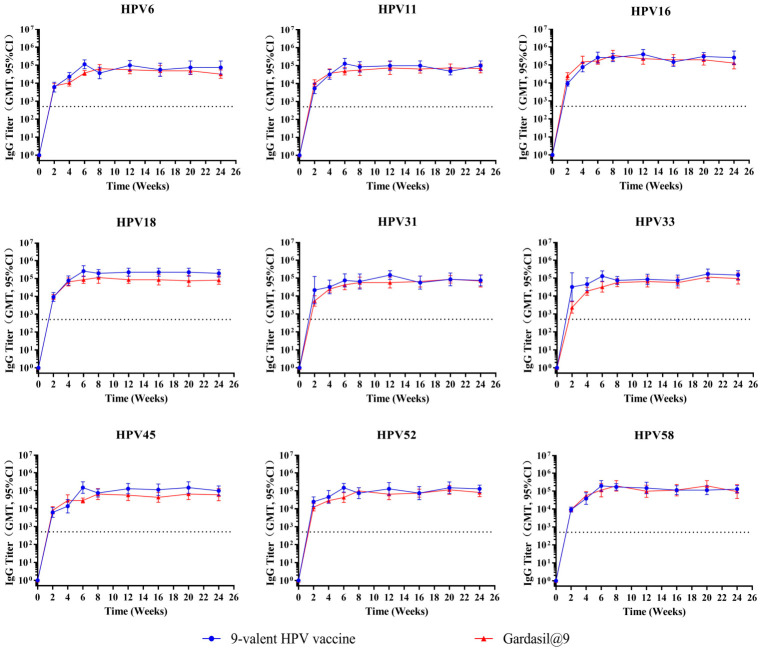
Dynamic changes in geometric mean titer of binding antibodies following a single immunization in mice. Female BALB/c mice (n = 10 per group) were immunized one time intramuscularly with 1/20 human dose of the test 9-valent HPV vaccine or Gardasil^®^9. Serum HPV type-specific IgG titers were monitored every 2 weeks for 24 weeks post-immunization. Error bars represent a 95% confidence interval, and the dashed line represents the threshold for positive conversion of binding antibodies (500).

**Figure 7:**
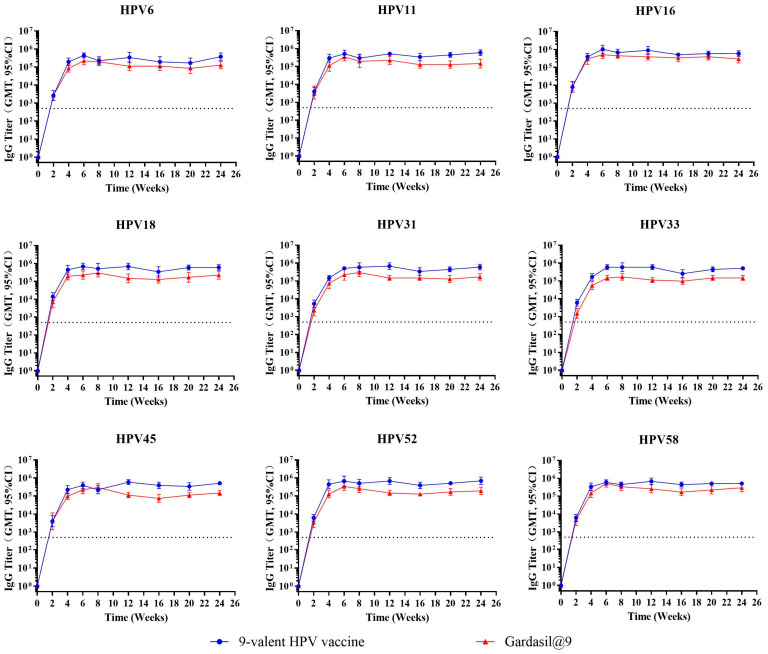
Dynamic changes in geometric mean titer of binding antibodies following three immunizations in mice. Female BALB/c mice (n = 10 per group) were immunized intramuscularly three times at 2-week intervals with 1/20 human dose of the test 9-valent HPV vaccine or Gardasil^®^9. Serum HPV type-specific IgG titers were monitored every 2 weeks for 24 weeks post-primary immunization. Error bars represent a 95% confidence interval, and the dashed line represents the threshold for positive conversion of binding antibodies (500).

**Figure 8:**
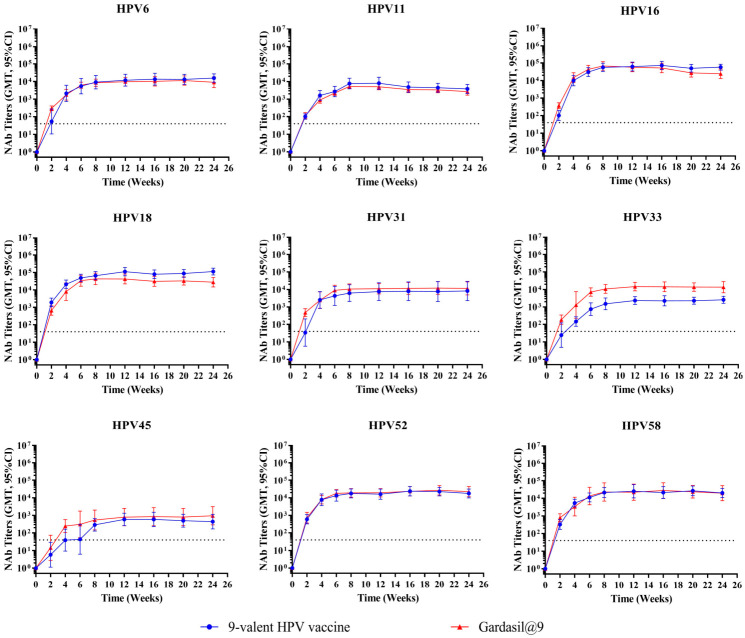
Dynamic changes in geometric mean titer of neutralizing antibodies following a single immunization in mice. Female BALB/c mice (n = 10 per group) were immunized one time intramuscularly with 1/20 human dose of the test 9-valent HPV vaccine or Gardasil^®^9. Serum neutralizing antibody titers were monitored every 2 weeks for 24 weeks post-immunization. Error bars represent a 95% confidence interval, and the dashed line represents the threshold for positive conversion of antibodies (40). NAb, neutralizing antibody.

**Figure 9:**
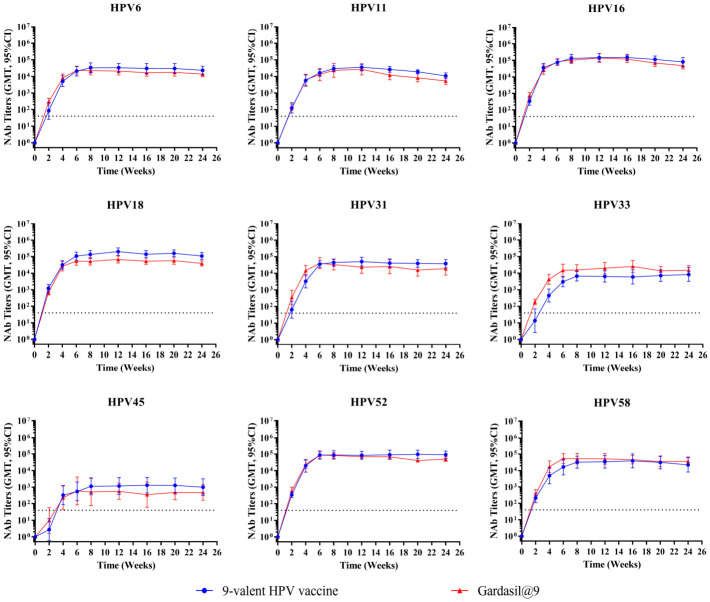
Dynamic changes in geometric mean titer of neutralizing antibodies following three immunizations in mice. Female BALB/c mice (n = 10 per group) were immunized intramuscularly three times at 2-week intervals with 1/20 human dose of the test 9-valent HPV vaccine or Gardasil^®^9. Serum neutralizing antibody titers were monitored every 2 weeks for 24 weeks post-immunization. Error bars represent a 95% confidence interval, and the dashed line represents the threshold for positive conversion of antibodies (40). NAb, neutralizing antibody.

To assess the persistence of HPV-specific immune responses induced by the test vaccine, BALB/c mice (n = 10 per group) were immunized intramuscularly with 1/20 human dose of the test 9-valent HPV vaccine or Gardasil^®^9. Mice received either one or three doses (administered at 2-week intervals), and serum binding antibody and neutralizing antibody titers were monitored at weeks 0, 2, 4, 6, 8, 12, 16, and 24. Temporal dynamics of binding antibody titers following one and three immunizations are depicted in Figure 6 and Figure 7, respectively. The neutralizing antibody‘s kinetic profiles are shown in Figure 8 and Figure 9 for one and three immunizations, respectively.

Following a single immunization, binding antibody titers (Figure 6) against all nine HPV types reached or approached peak levels at 6–8 weeks, with peak titers ranging from 10^4^ to 10^5^ or slightly above 10^5^. After reaching peak levels, binding antibody levels remained stable with no significant decrease through week 24. Administration of three immunizations (Figure 7) markedly enhanced the peak magnitude of binding antibody responses, with titers reaching 10^5^ to 10^6^ for all HPV types. After peaking at 6–8 weeks, binding antibody levels remained sustained without obvious reduction until week 24. The kinetic profiles and magnitude of binding antibody responses induced by the test vaccine were highly comparable to those of Gardasil^®^9 following both one and three immunization schedules.

No detectable HPV-specific IgG or neutralizing antibody titers were observed in the negative control groups throughout the 24-week observation period (Table S1).

Neutralizing antibody analyses showed that after a single immunization (Figure 8), type-specific neutralizing antibody titers peaked at 6–8 weeks and remained stable through week 24. Peak neutralizing antibody titers reached 10^4^–10^5^ for HPV16, 18, 52, and 58; 10^3^–10^4^ for HPV6, 11, 31, and 33; and approximately 10^3^ for HPV45. The test vaccine displayed immune persistence profiles similar to Gardasil^®^9. Geometric mean titers (GMTs) of neutralizing antibodies against HPV33 were slightly lower for the test vaccine relative to the positive control. The neutralizing antibody GMTs for the other eight HPV types were comparable between the test vaccine and Gardasil^®^9.

Three immunizations (Figure 9) elicited a significant increase in neutralizing antibody levels compared with a single dose. Peak neutralizing antibody titers for HPV16, 18, and 52 exceeded 10^5^, equivalent to those induced by Gardasil^®^9. Peak GMTs of neutralizing antibodies against HPV6, 11, 31, and 58 reached 10^4^–10^5^, comparable to the positive control. Peak neutralizing antibody GMTs for HPV33 reached 10^3^–10^4^, while those for HPV45 exceeded 10^3^ (slightly higher than the corresponding response induced by Gardasil^®^9 which approached 10^3^). Neutralizing antibody levels remained stable from 6 to 8 weeks through week 24, with kinetic dynamics consistent with those of the positive control.

### 3.3. The 9-Valent HPV Vaccine Induced High-Level and Durable Immune Responses in Rats

The immunogenicity and dose effects of the test 9-valent HPV vaccine were further evaluated in rats. Female Wistar rats (8–9 weeks old; 180–220 g in weight; n = 10 per group) were intramuscularly administered with three inoculations at 2-week intervals, with three dose groups: high (1/10), medium (1/20), and low (1/40) doses. The licensed Gardasil^®^9 vaccine at a 1/20 dose served as the positive control. Serum samples were collected 2 weeks post-third immunization for HPV type-specific neutralizing antibody titer analysis using a pseudovirus-based assay.

**Figure 10:**
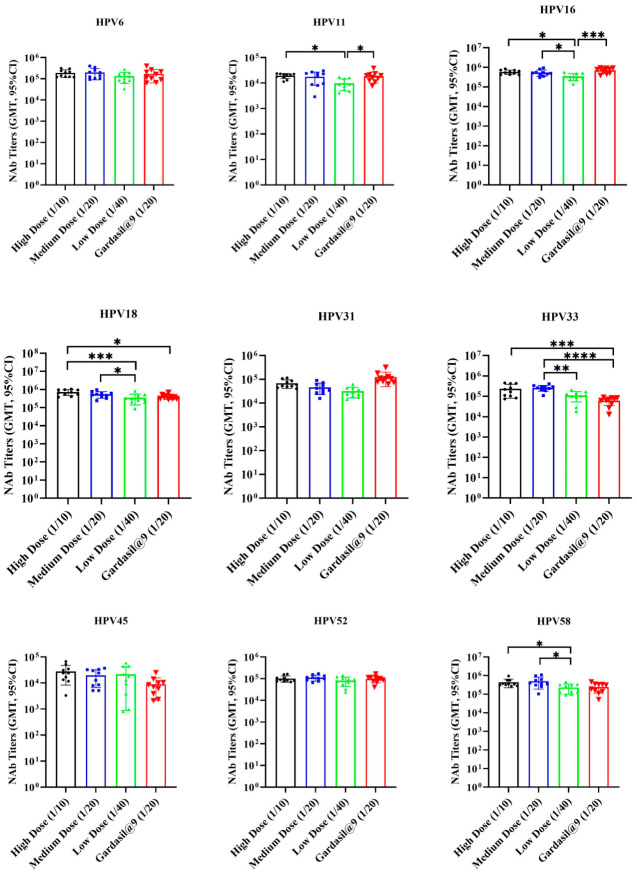
The neutralizing antibody titers in serum 2 weeks after the third vaccination in rats. Wistar rats were inoculated three times with 1/10, 1/20, or 1/40 dose of the test 9-valent HPV vaccine, with 1/20 dose of Gardasil@9 as positive control. NAb, neutralizing antibody. *, *p* < 0.05; **, *p* < 0.01; ***, *p* < 0.001; ****, *p* < 0.0001.

Two weeks after the third vaccination (Figure 10), GMTs of neutralizing antibodies against HPV 6, 11, 16, 18, 31, 33, 52, and 58 reached 10^3^–10^4^ or higher, whereas GMTs for HPV 45 reached 10^2^–10^3^. When comparing the medium-dose (1/20) group to Gardasil^®^9 (1/20), neutralizing antibody titers specific for HPV 6, 11, 16, 18, 31, 45, 52, and 58 were equivalent between the two groups. For HPV 33, the 9-valent HPV vaccine induced significantly higher titers than the Gardasil^®^9 group (*p* < 0.001).

For dose effects, neutralizing antibody titers for HPV6, 31, 45, and 52 showed no significant differences among the three dose groups. For HPV11, the high-dose (1/10) group had higher titers than the low-dose group (*p* = 0.0104), equivalent to the medium-dose group, with no significant difference between the medium- and low-dose groups. For HPV16, 18, and 58, both the high- and medium-dose groups had higher titers than the low-dose group (*p* = 0.0033 and 0.0172 for HPV16; *p* = 0.0008 and 0.0421 for HPV18; *p* = 0.0465 and 0.0338 for HPV58), with no significant difference between the high- and medium-dose groups. For HPV33, the medium-dose group had higher titers than the low-dose group (*p* = 0.0057) and was equivalent to the high-dose group; however, the high-dose group showed no significant difference from the low-dose group. When analyzing GMTs of neutralizing antibody titers, the high-dose group was equivalent to the medium-dose group and higher than the low-dose group for all nine HPV types.

To assess immune durability, female Wistar rats (8–9 weeks old; 180–220 g in weight; n = 10 per group) received either a single inoculation or three inoculations at 2-week intervals, with three dose groups for each immunization schedule: high (1/10), medium (1/20), and low (1/40) doses. Gardasil^®^9 administered at 1/20 of the human dose was set as the positive control. Antibody persistence was monitored at weeks 2, 4, 6, 8, 12, and 16.

**Figure 11:**
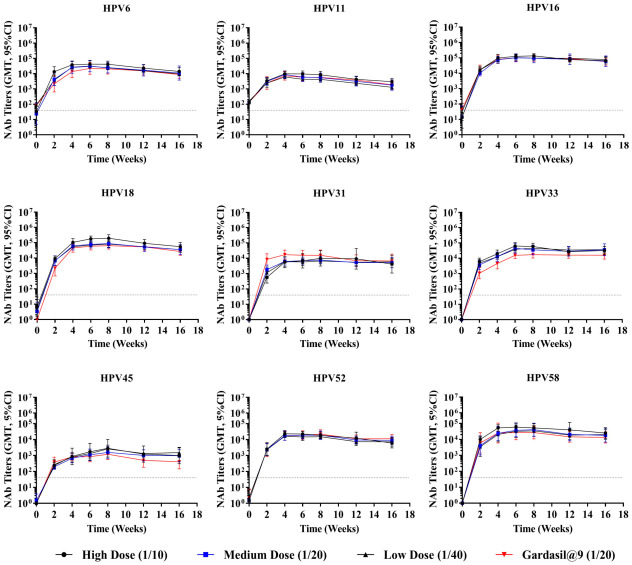
Dynamic changes in geometric mean titers of neutralizing antibodies after a single inoculation in Wistar rats. Female Wistar rats (n = 10 per group) received single inoculation intramuscularly, with three dose groups: high (1/10), medium (1/20), and low (1/40) dose. Gardasil^®^9 administered at 1/20 of the human dose was set as the positive control. Serum neutralizing antibody titers were monitored every 2 weeks up to week 16. Error bars represent a 95% confidence interval. The dashed line represents the threshold for positive conversion of antibodies (40). NAb, neutralizing antibody.

Neutralizing antibody titers for the single-dose groups (Figure 11) reached or approached peak values 4–6 weeks post-vaccination, with GMTs for HPV6, 16, 18, 52, and 58 reaching 10^4^–10^5^ and those for HPV11, 31, 33, and 45 reaching 10^3^–10^4^. After reaching peak values, titers began to decrease slowly; by week 16, HPV6, 16, 18, 52, and 58 maintained GMTs of approximately 10^4^ or higher, and HPV11, 31, 33, and 45 maintained levels around 10^3^–10^4^.

**Figure 12:**
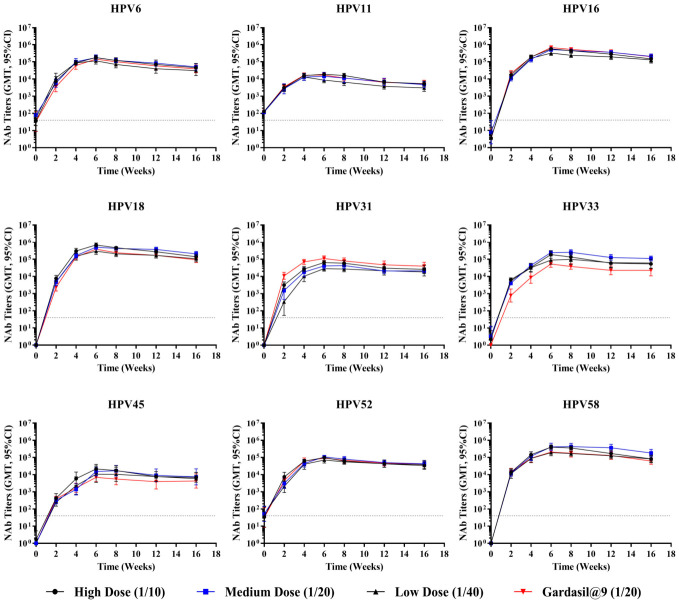
Dynamic changes in geometric mean titers of neutralizing antibodies following three inoculations in rats. Female Wistar rats (n = 10 per group) received three inoculations intramuscularly at 2-week intervals, with three dose groups: high (1/10), medium (1/20), and low (1/40) dose. Gardasil^®^9 administered at 1/20 of the human dose was set as the positive control. Serum neutralizing antibody titers were monitored every 2 weeks up to week 16. Error bars represent a 95% confidence interval. The dashed line represents the threshold for positive conversion of antibodies (40). NAb, neutralizing antibody.

Neutralizing antibody results from the three-dose groups (Figure 12) showed that all antigen types of the 9-valent HPV vaccine had strong immunogenicity in rats. After two booster immunizations, there was a noticeable increase in the rats’ neutralizing antibody levels compared to single-dose immunization. Neutralizing antibody titers peaked 2 weeks after the third immunization (week 6). Across various dose groups for HPV types 6, 16, 18, and 58, maximum titers reached 10^5^–10^6^; for HPV types 11, 31, 45, and 52, as well as the high-dose group of HPV type 33, peak titers were 10^4^–10^5^; while medium- and low-dose groups of HPV type 33 achieved peak titers of 10^3^–10^4^. Following the peak, antibody titers began to decline gradually. By 12 weeks after the third immunization (week 16), neutralizing antibody titers had decreased to approximately one-half to one-third of their peak levels.

Both one- and three-inoculation regimens of the 9-valent HPV vaccine exhibited consistent trends in the induction of neutralizing antibodies, and showed similar differences in comparison with Gardasil^®^9. In comparison with the positive control (Gardasil^®^9), the test 9-valent HPV vaccine elicited comparable neutralizing antibody responses against eight HPV types (6, 11, 16, 18, 33, 45, 52, and 58) throughout the 16-week observation period. The difference in HPV31-specific antibody titers between the test vaccine and the positive control gradually narrowed over time. By week 8, HPV31 antibody levels had become comparable to those of the positive control and remained at similar levels up to week 16.

HPV-specific neutralizing antibody titers were undetectable in the negative control groups throughout the entire 16-week observation period (Table S2).

## 4. Discussion

The key to developing HPV vaccines lies in the ability to produce HPV major capsid protein L1 VLPs in large quantities and with high efficiency. All currently marketed HPV vaccines utilize genetically engineered HPV L1 self-assembled VLPs as their core antigen component. These VLPs mimic the morphology and structure of genuine HPV, exhibiting strong immunogenicity [32,33,34,35,36]. The commonly used expression systems can be categorized into eukaryotic expression systems and prokaryotic expression systems. Widely used eukaryotic expression systems include yeast expression systems and insect baculovirus expression systems. HPV L1 proteins expressed in eukaryotic systems can self-assemble into HPV type-specific hollow structures, while these systems face challenges such as high production costs and low yields [37,38]. In prokaryotic expression systems, *Escherichia coli*-based production of HPV L1 protein exhibits distinct advantages over the *Saccharomyces cerevisiae* platform adopted by Gardasil^®^9 in production efficiency and cost economy. *E. coli* features fast proliferation, high-level recombinant protein yield and high-density fermentation feasibility, along with low-cost, simple culture medium requirements [39,40]. These merits greatly cut raw material, energy, labor and equipment costs, achieving high productivity and low unit production costs. Such differences lead to a notable price gap between *E. coli*- and yeast-derived HPV vaccines. For instance, the *E. coli*-based 9-valent HPV vaccine Cecolin^®^9 is priced at 499 RMB per dose in China, approximately 60% lower than Gardasil^®^9 (1300 RMB per dose) [41]. Most of the HPV L1 protein expressed in *E. coli* loses its natural conformation and tends to form inclusion bodies. Although VLPs can be obtained through inclusion body purification and refolding steps, significant protein loss occurs during the refolding process, resulting in low yields [42,43,44]. Professor Chen Xiaojiang discovered that truncating the N-terminus by X amino acids (0 < X < 10) could significantly enhance the expression level of the HPV16 L1 protein. Similarly, truncating the C-terminus by Y amino acids (0 < Y < 31) increased the proportion of correctly folded soluble L1 pentamers while reducing the formation of disordered L1 polymers [32,45]. Based on these findings, we achieved increased soluble and stable L1 protein expression in *E. coli* by truncating the N-terminus and C-terminus, enabling the formation of more correctly folded pentameric L1 proteins and minimizing the formation of polymeric structures. As a result, during expression and purification, the L1 protein primarily existed as soluble pentamers, without requiring in vitro folding that is necessary for other *E. coli*-produced HPV vaccines, which was the main advantage to other vaccines. In this study, DLS analysis of the purified proteins revealed a particle size distribution centered around 12 nm, which is characteristic of typical L1 pentamers. This not only simplified the purification process and increased both purification yield and total production yield but also significantly enhanced product quality and reduced production costs, making the tested 9-valent HPV vaccine a more economically viable candidate.

The particle size of VLPs is one of the critical factors in effectively stimulating the human immune system, particularly humoral immunity to produce neutralizing antibodies [46]. This is because their size resembles that of natural viruses, making them easily recognizable and phagocytosed by antigen-presenting cells. The particle sizes of VLPs in different HPV vaccines were very similar, falling within the range of 50–60 nm [47]. This is highly consistent with the particle size of native HPV viruses (approximately 55 nm). Under cryo-electron microscopy, we observed purified HPV types 6, 11, 16, 18, 31, 33, 45, 52, and 58 L1 VLPs, which were uniformly distributed and measured approximately 50–60 nm in size. The morphology and size of all VLP types were highly similar to those of natural HPV virus particles. Immunogenicity and immune persistence tests in mice and rats demonstrated that our product can induce an immune response against HPV, with effects comparable to those of similar products on the market.

The binding antibody levels across the nine types are similar, while the neutralizing antibody levels vary among different types. Specifically, the neutralizing antibody titers for HPV11 and HPV45 are lower than those for other types in both mice and rats. Studies on the 9-valent vaccine from Shanghai Zerun Biotech Co., Ltd. also show the same trend in mice, rats, and monkeys [48]. This may be attributed to the low immunogenicity of the L1 for these two HPV types.

Although preclinical data have verified that this 9-valent HPV vaccine confers immune efficacy comparable to Gardasil^®^9, human clinical trials are still required to systematically evaluate its clinical immunogenicity, efficacy and safety in target populations. Clinical validation constitutes a mandatory regulatory prerequisite for vaccine licensure, and to confirm that the cost benefits of the *E. coli* expression system do not impair protective potency, supporting its use as an affordable alternative HPV vaccine to broaden population prevention accessibility. This candidate vaccine is currently in a Phase III clinical trial in China (CTR20201791), which will provide solid clinical evidence for its clinical application and public health value.

There are limitations in our study. Firstly, it was not investigated whether the antibodies induced by our 9-valent HPV VLP vaccine could neutralize HPV types beyond these nine included in the vaccine. We planned to test the cross-reactivity of this 9-valent HPV vaccine against other HPV types, such as HPV types 35, 52, 56 and 68. Secondly, no typical dose–effect difference in immunogenicity was observed among the high-, medium-, and low-dose groups in rats. This may be attributed to the strong immunogenicity of the antigen, such that even the low dose used in this study reached a saturation level, or the twofold dose difference was insufficient to elicit a statistically significant difference in immune response. In subsequent studies, the antigen dose could be further reduced, and the fold difference between doses could be increased to explore the appropriate dose range that demonstrates a clear dose–response relationship between the antigen dose and immune effect.

The test 9-valent HPV vaccine elicited high and durable immune responses in both mice and rats, which provides strong support for its further clinical development. This vaccine is expected to serve as a viable alternative to currently marketed vaccines and help alleviate the global supply shortage of 9-valent HPV vaccines.

## 5. Conclusions

The candidate 9-valent HPV vaccine elicited robust and long-lasting immune responses comparable to those induced by the commercial Gardasil^®^9 vaccine, providing strong preclinical evidence for its further clinical translation. These preclinical data support the further clinical development of this 9-valent HPV vaccine candidate, which holds great promise as a valuable alternative to currently marketed products and may help alleviate the global supply shortage of 9-valent HPV vaccines and contribute to global HPV prevention.

## Figures and Tables

**Table 1:** The average sizes and PdI values of pentamers and VLPs detected by DLS.

HPV Type	Pentamer		VLP	
	Average Size (nm)	PdI	Average Size (nm)	PdI
HPV6	16.7	0.213	49.39	0.019
HPV11	12.71	0.033	53.24	0.021
HPV16	12.25	0.036	47.5	0.024
HPV18	12.12	0.058	56.46	0.019
HPV31	12.21	0.031	61.21	0.078
HPV33	12.62	0.158	48.44	0.012
HPV45	12.14	0.026	50.98	0.044
HPV52	12.26	0.099	62.2	0.026
HPV58	12.53	0.037	52.54	0.02

PdI, polydispersity index. This parameter reflects the heterogeneity of particle size distribution. A lower PdI value indicates a more uniform particle population. A PdI value < 0.1 is generally considered the criterion for monodisperse particles.

## Data Availability

The data presented in this study are available on request from the corresponding authors.

## Supplementary Materials

The following supporting information can be downloaded at: https://www.mdpi.com/article/10.3390/vaccines14050407/s1, Figure S1: The original SDS-PAGE figures. The full blots for Figure 1b,d,g were accidentally overwritten by cropped files during saving. Table S1: Antibody titers of the negative control mice group. Table S2: Antibody titers of the negative control rat group.
