# Supplementary material for: Immunogenicity of an Escherichia coli-Produced Recombinant 9-Valent Human Papillomavirus Vaccine in Mice and Rats
Source: Vaccines (Basel). 2026 May 1;14(5):407. doi: 10.3390/vaccines14050407 (PMC13211545; doi:10.3390/vaccines14050407)
Supplement: Supplementary file 1 [file vaccines-14-00407-s001.zip › vaccines-4244409-supplementary.pdf]

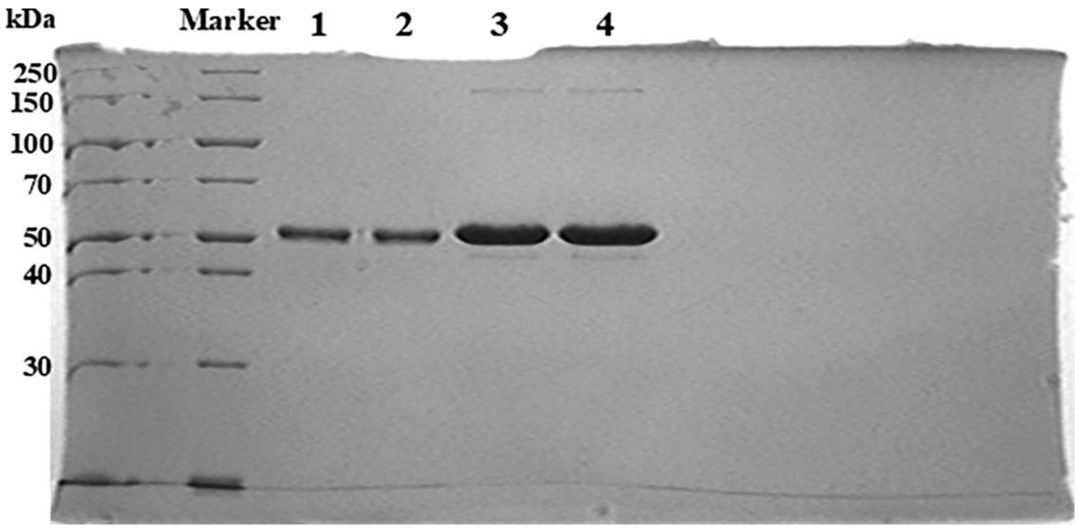

Figure 1a

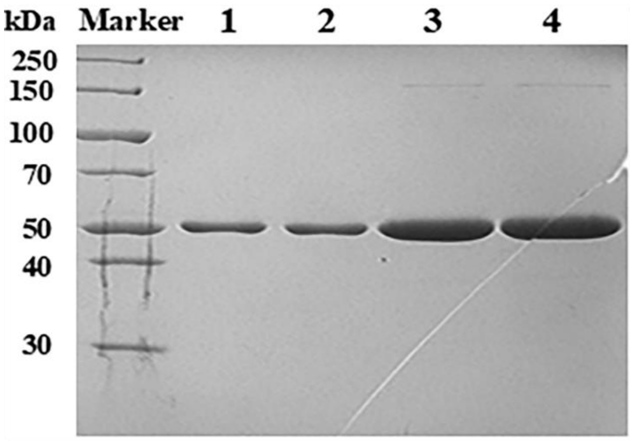

Figure 1b

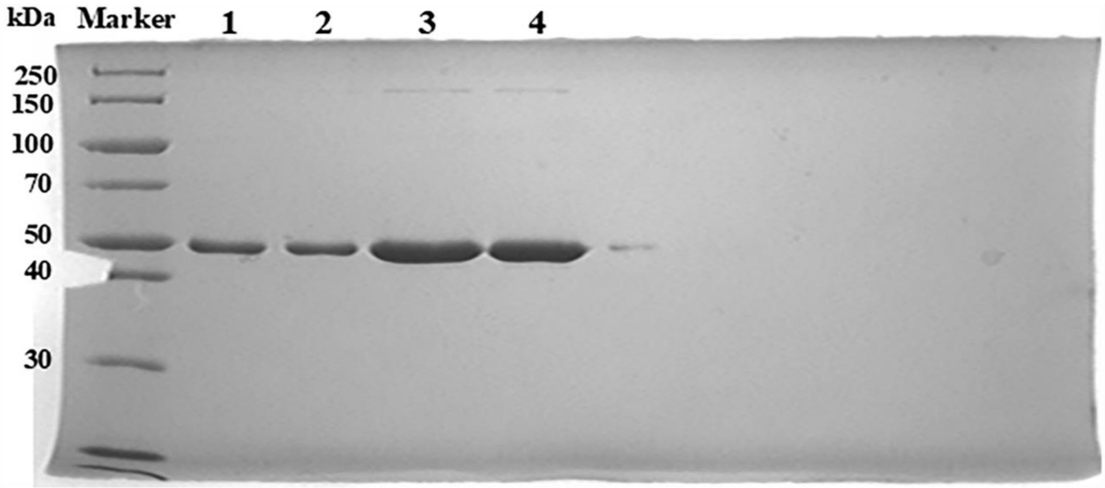

Figure 1c

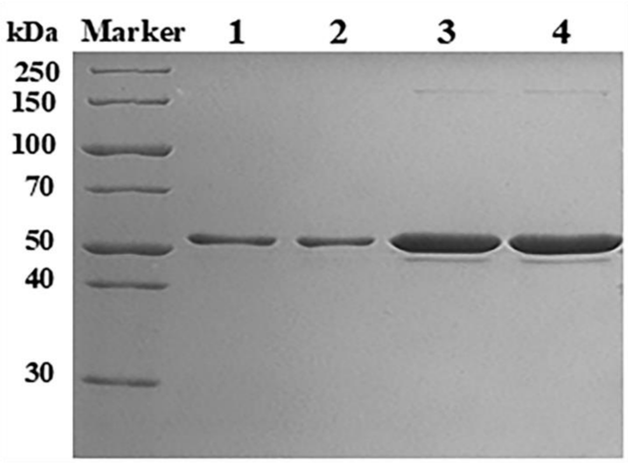

Figure 1d

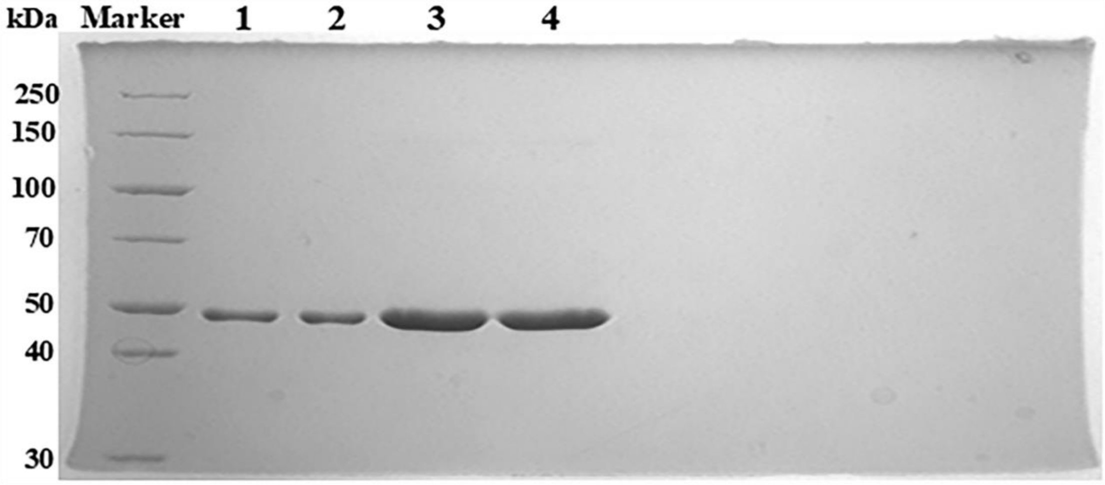

Figure 1e

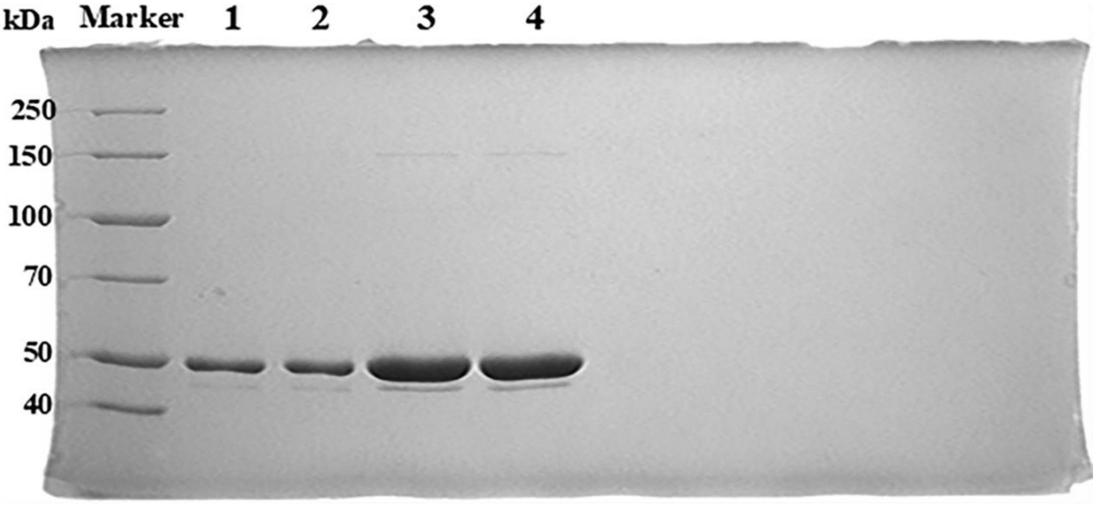

Figure 1f

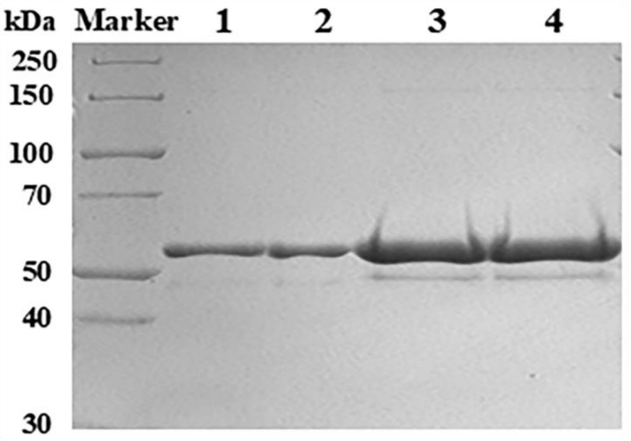

Figure 1g

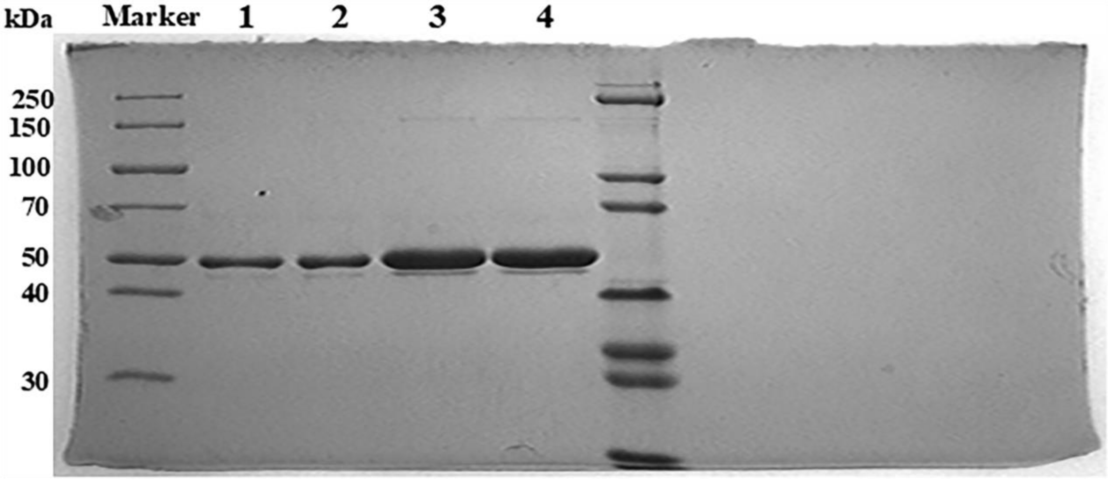

Figure 1h

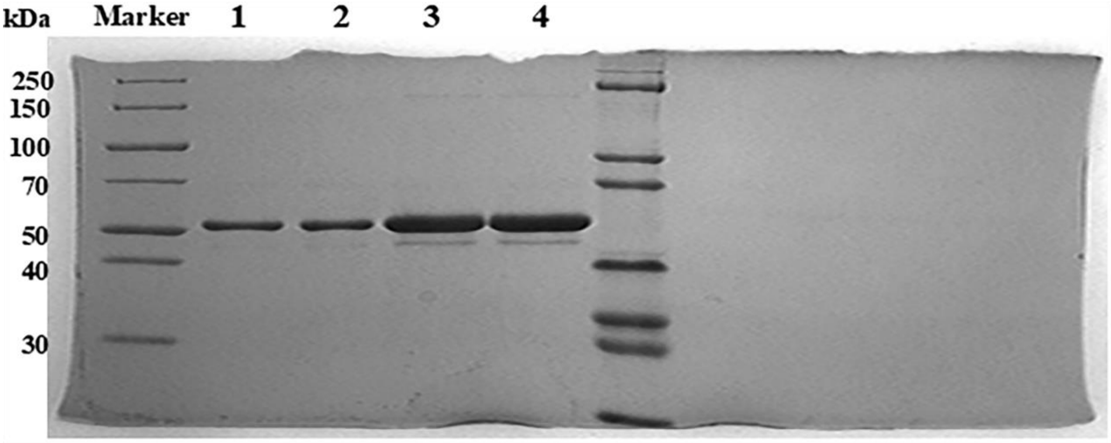

Figure 1i

Figure S1. The original SDS-PAGE figures. The full blots for Figure 1b, 1d and 1g were accidentally overwritten by cropped files during saving.

Table S1. Antibody titers of the negative control mice group

Antibody titers	week 2	week 4	week 6	week 8	week 12	week 16	week 20	week 24
IgG titers	<500	<500	<500	<500	<500	<500	<500	<500
NAb titers	<40	<40	<40	<40	<40	<40	<40	<40

NAb, neutralizing antibody.

Table S2. Antibody titers of the negative control rat group

Antibody titers	week 2	week 4	week 6	week 8	week 12	week 16
NAb titers	<40	<40	<40	<40	<40	<40

NAb, neutralizing antibody.
